# Choroidal and Retinal Imaging Biomarkers in Different Types of Macular Neovascularization

**DOI:** 10.3390/jcm12031140

**Published:** 2023-02-01

**Authors:** Lisa Toto, Maria Ludovica Ruggeri, Federica Evangelista, Chiara Trivigno, Rossella D’Aloisio, Chiara De Nicola, Pasquale Viggiano, Emanuele Doronzo, Marta Di Nicola, Annamaria Porreca, Rodolfo Mastropasqua

**Affiliations:** 1 Ophthalmology Clinic, Department of Medicine and Science of Ageing, University G. D’Annunzio Chieti-Pescara, via dei Vestini 31, 66100 Chieti, Italy; 2 Ente Ecclesiastico Ospedale Generale Regionale, “F.Miulli”, Strada Provinciale Acquaviva-Santeramo Km 4100, 70021 Acquaviva delle Fonti, Italy; 3 Department of Basic Medical Science, Neuroscience and Sense Organs, University of Bari “Aldo Moro”, 70121 Bari, Italy; 4 Laboratory of Biostatistics, Department of Medical, Oral and Biotechnological Sciences, University “G. d’Annunzio” Chieti-Pescara, via dei Vestini 31, 66100 Chieti, Italy

**Keywords:** macular neovascularization, optical coherence tomography angiography, spectral-domain optical coherence tomography

## Abstract

Background: The aim of this study was to investigate optical coherence tomography (OCT) and OCT angiography (OCTA) parameters in patients with neovascular age-related macular degeneration (nAMD) and macular neovascularization (MNV) type 1, type 2, and type 3. Methods: In this retrospective study, 105 treatment-naïve eyes of 105 patients (60 men and 45 women) with a definite diagnosis of active nAMD and MNV of different types and 105 frequency-matched age and gender healthy subjects were evaluated (61 men and 44 women). All subjects underwent a full ophthalmic examination and multimodal imaging assessment, including spectral domain (SD) OCT and OCTA. The main outcome measures were choroidal vascularity index (CVI), subfoveal choroidal thickness (SFCT), central macular thickness (CMT), and outer retina to choriocapillaris (ORCC) MNV flow area (ORCCFA). Results: Significant differences were found in terms of CVI, CMT, and ORCCFA between MNV 1 and the two other groups. CVI was significantly different between MNV 1 and healthy control patients (*p* < 0.001) and between MNV 1 and MNV 2 (*p* < 0.001). ORCCFA and CMT were significantly different between MNV1 and MNV2 (*p* < 0.005). The difference in subfoveal CT between the three groups was not statistically significant (*p* = 0.458). A significant negative correlation was found between CVI and ORCCFA. Furthermore, CVI showed a positive correlation with subfoveal CT.

## 1. Introduction

The choroid plays an important role in the pathogenesis of multiple chorioretinal diseases, including age-related macular degeneration (AMD) [1,2,3]. Histological and in vivo studies have shown a reduction in choroidal vessel diameter and choriocapillary density in AMD, suggesting a possible choroidal role in AMD [4,5]. It is useful to study the choroid in order to better understand disease pathogenesis, progression, and possible therapeutic effects.

Advances in optical coherence tomography (OCT) technology and the gradual shift from manual to semi-automatic segmentation and binarization methods have provided more accurate and reproducible choroidal parameters [6,7,8,9,10]. In this regard, choroidal vascularity index (CVI) is emerging as a new and relatively robust imaging tool that refers to the ratio of the luminal area to the cross-sectional choroidal area, representing a quantitative parameter that can help the assessment of choroidal vasculature. 

CVI alteration may be representative for choroidal vasculature impairment and therefore may play a central role in monitoring the disease progression, patient follow-up, and response to the therapy [11,12,13,14].

Several studies have shown that CVI is less variable than choroidal thickness (CT), being influenced by fewer confounding factors. CVI is more sensitive and specific than manual and linear CT, providing information about disease morphology, assessing both stromal and vascular choroidal components [15,16].

A few recent studies have shown that AMD eyes have significantly lower CVI compared to healthy eyes, both in wet and dry forms [17,18].

In early and intermediate AMD, Corvi et al. found a reduced CVI in eyes with drusen, suggesting a possible role of vascular depletion and fibrotic replacement [19].

Giannaccare et al. showed a decreased CVI in geographic atrophy compared to healthy controls [20].

In neovascular AMD (nAMD), CVI was significantly reduced, despite an unchanged CT, probably due to an increased stromal component and decreased vascular area [21].

Invernizzi and colleagues performed a longitudinal CVI analysis from baseline dry or inactive nAMD, showing a change in CVI values from inactive to active disease in type 1 neovascularization (NV) [22].

Larger studies are required to validate CVI as a new surrogate marker for clinical trials, considering that CVI studies mainly concern small samples and Asian populations, and also considering that studies on all types of AMD macular neovascularization (MNV) are rare [23].

Our study aimed to analyze CVI in patients with AMD and MNV type 1, type 2, and type 3 and correlate CVI of different MNV types, OCT parameters, and OCT angiography (OCTA) MNV flow parameters. 

## 2. Materials and Methods

### 2.1. Study Participants

In this retrospective study, 105 treatment-naïve eyes of 105 patients (60 men and 45 women) with a definite diagnosis of nAMD with active MNV and 105 frequency-matched age and gender-healthy subjects were evaluated (61 men and 44 women). 

MNV was defined and classified according to the Consensus Nomenclature for Reporting.

Neovascular age-related macular degeneration data were used to define all forms of neovascularizations, including NV originating from the retinal circulation toward the outer retina, described as type 3 MNV. In contrast, the definition of type 1 MNV and type 2 MNV was used to define the proliferation of new vessels, respectively, from the choriocapillaris into the sub RPE space (type 1) and in the subretinal space (type 2). The term mixed type 1 and type 2 NV can be applied to define the presence of new vessels in the subretinal and sub-RPE compartments [24].

The enrolled patients were referred to the retina center of the Ophthalmology Clinic of University “G. d’Annunzio”, Chieti-Pescara, Italy, between October 2020 to June 2021. 

The study adhered to the tenets of the Declaration of Helsinki. Informed consent was obtained from all patients. 

Inclusion criteria for all AMD cohorts were as follows: (1) patients aged ≥ 50 years; (2) active naïve nAMD. Exclusion criteria were as follows: (1) previous treatments for MNV, such as photodynamic therapy and intravitreal injections of anti-vascular endothelial growth factor (VEGF); (2) history of other chorioretinal disorders; (3) no relevant media opacities to ensure adequate image quality. If both eyes had features in line with inclusion criteria, both were included in the study cohort.

All subjects underwent a full ophthalmic examination using a multimodal imaging approach. Spectral domain -OCT (SD-OCT), fluorescein angiography (FA), indocyanine green angiography (ICGA), and OCT angiography (OCTA) were performed using Spectralis HRA + OCT (Heidelberg Engineering, Heidelberg, Germany). OCTA for flow area was obtained using RTVue XR Avanti OCT-A system (AngioVue system, version 2018.1.0.43, Optovue^®®^ Inc., Fremont, CA, USA). 

Main outcome measures were as follows: central macular thickness (CMT), subfoveal choroidal thickness (CT), CVI, and outer retina to choriocapillaris (ORCC) MNV flow area (ORCCFA).

### 2.2. Imaging Protocol

The acquisition protocol for SD OCT included 49 horizontal raster dense linear B-scans centered on the fovea. A horizontal and vertical B-scans centered on the fovea with enhanced depth imaging (EDI) mode were acquired for all patients. 

Central macular thickness was measured using the central 1 mm diameter circle of the ETDRS thickness map.

Subfoveal choroidal thickness measured vertically from the outer border of the RPE to the inner border of the sclera was measured using the inbuilt manual caliper on EDI OCT scans.

All measurements were performed by two independent experienced readers. 

### 2.3. OCT Analysis

**Figure 1:**
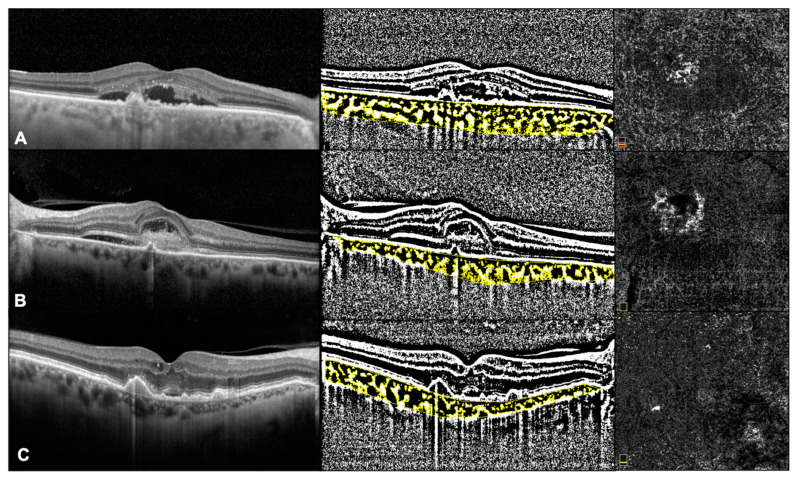
OCT and OCTa images in Type 1 MNV (**A**), type 2 MNV (**B**), and type 3 MNV (**C**).

CVI was investigated using a previously reported algorithm [12]. In brief, we exported the EDI-OCT horizontal and vertical single line scan passing through the fovea. Afterwards, the image analysis involved the automated binarization of a linear OCT B-scan after delimiting the choroidal boundaries. The choroid-EPR junction and the sclero-choroidal junction were considered as the limits of the ROI. Total choroidal area (TCA) was calculated as the total area of the ROI. The images were binarized using Niblack’s auto local threshold, and dark pixels were defined as the luminal choroidal area (LCA), while white pixels were defined as stromal choroidal area (SCA). CVI was obtained as the ratio between LCA and TCA (Figure 1).

### 2.4. OCTA Analysis

In all cases, 3 × 3 mm volume scans were performed in all eyes, and only when the scan did not cover the entire lesion a 6 × 6 mm scan was acquired. 

MNV flow area was calculated in all cases. The ORCC slab was set by manually extending from the outer boundary of the outer plexiform layer to 8 µm beneath Bruch’s membrane, and projection artifact removal algorithms were also used.

Images were exported as JPEG files and then analyzed with Image J software version 1.52° (National Institutes of Health, Bethesda, MD, USA; available at http://rsb.info.nih.gov/jj/index.html (accessed on June 2021). The NV lesions were manually circumscribed by two independent retinal specialists, and the flow within was calculated as the number of pixels over a nonperfusion threshold, then converted in a comparable mm^2^ area value (Figure 1). 

NV density was considered as the ratio of pixels occupied by flowing vasculature to all pixels included in the analyzed region, as previously described [25,26].

### 2.5. Statistical Analysis

Descriptive statistics were produced according to the distribution of variables. For continuous variables, the median, first (q1), and third quartile (q3) are presented; for categorical variables, the absolute frequency (n) and column percentage (%) are reported. Lin’s concordance correlation coefficient (CCC) was applied to evaluate the magnitude of bias between readers with its 95% confidence interval. According to high CCC values, each subject was attributed the mean value between reader 1 and reader 2. The correlation analysis was developed through the network correlation analysis using Spearman’s correlation coefficient (rho) as the weight of the edges. The association between categorial variables was assessed using the Chi-square test. The Kruskal–Wallis test was performed to detect if there were differences in the value of CVI among MNV1, MNV2, MNV3, and healthy patients and if there were differences in the values of CMT, subfoveal CT, and ORCCFA. Post-hoc analysis was performed using the Mann–Whitney U test. All statistical tests were two-sided, with a significance level set at *p* ≤ 0.05. Statistical analysis was performed using R software environment for statistical computing and graphics version 3.5.2 (R Foundation for Statistical Computing, Vienna, Austria; https://www.R-project.org/ accessed on June 2021).

## 3. Results

### 3.1. Characteristics of Patients Included in the Analysis

The demographic characteristics of the patients are shown in Table 1. The mean age of the 105 healthy controls (61 men and 44 women) was 75.5 [70.0;81.0] years.

Among enrolled patients, 37 eyes showed type 1 MNV, 52 eyes showed type 2 MNV, and 16 eyes showed type 3 MNV. No differences were found between the three groups for age and gender.

### 3.2. SD-OCT and OCTA Parameters

**Figure 2:**
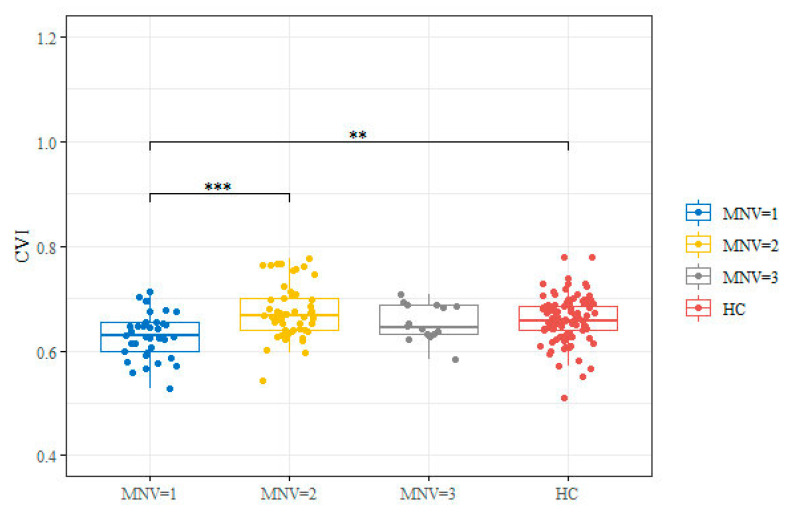
Box and whisker plot for CVI value by groups (MNV = 1, 2, 3 and HC). Box and whisker plots show 25th and 75th percentile ranges (box) with 95% confidence intervals (whiskers) and median values (transverse lines in box). Significance code: ** *p*-value < 0.01; *** *p*-value < 0.001 derived from Wilcoxon rank sum test post hoc. HC: healthy controls.

SD OCT and OCTA images were acceptable for all eyes, and quantitative parameters were scored in all cases. At baseline, significant differences were found in terms of CVI, CMT, and ORCCFA between MNV 1 and the two other groups (Table 2). Furthermore, there was a significant difference in terms of CVI between MNV 1 and the healthy control group (*p* < 0.001) and between MNV 1 and MNV 2 (*p* < 0.001) (Figure 2). ORCCFA and CMT were significantly different between MNV1 and MNV2 (*p* < 0.005). The differences in subfoveal CT between the three groups were not statistically significant (*p* = 0.458). 

### 3.3. Correlation Network Analysis for MNV Types

**Figure 3:**
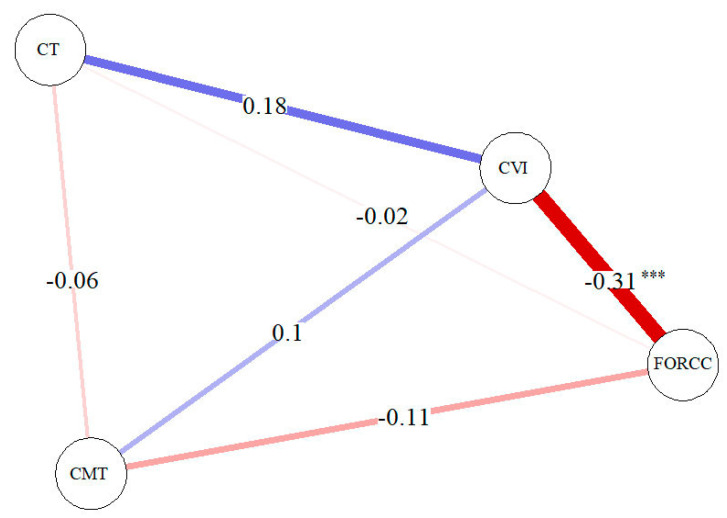
Correlation network analysis for MNV (all types). A blue line shows a positive correlation and a red line shows s negative correlation. The strength of the relation is shown by the thickness of the line. Significance code: *** *p*-value < 0.001.

Interestingly, a significant negative correlation was found between CVI and ORCCFA (Figure 3) considering all MNV types. Furthermore, CVI showed a positive correlation with CT.

## 4. Discussion

In the present study, we examined the retinal and choroidal parameters by means of OCT and OCTA in eyes affected by different naïve active MNV types. Interestingly, we found significant CVI differences between MNV 1 and healthy controls (*p* < 0.001) and between MNV 1 and MNV 2 (*p* < 0.001), with MNV1 CVI values being lower than the CVI values of the control group and MNV2 group. Furthermore, CVI was significantly negatively related with ORCCFA and positively related with CT. 

Impairment of CVI in eyes with AMD has been previously reported in different studies. Velaga et al. and Viggiano et al. highlighted the reduced CVI in eyes with AMD with different drusen patterns compared with healthy eyes, with the choroidal vasculature being more altered in patients with reticular pseudodrusen (RPD) compared to patients with non-neovascular AMD without RPD [18,27]. 

Likewise, Koh et al. found that eyes with treatment-naïve AMD, both dry and wet, were characterized by a reduced volume of the choroidal vasculature on a two-dimensional scan compared to the normal controls [28]. 

These observations are very similar to our findings showing significant lower CVI in eyes affected by MNV1 compared to the healthy group. In addition, in our current analysis, we also found significant differences between MNV subgroups, with results showing that eyes with MNV type 1 had a lower CVI compared with MNV type 2 eyes. 

These results confirm, as previously suggested, a role of underlying choroidal ischemia or reduced choroidal vascularity in the pathogenesis of the MNV. 

Moreover, we correlated the CVI value with ORCCFA, showing a significant negative correlation for all MNV types. We were also able to support the hypothesis that active type MNV takes blood from the choroidal vessels. 

Other authors have described choroidal changes, and particularly CT and CVI, in relation to active disease in nAMD eyes in order to predict NV development or recurrence before they are otherwise evident clinically. Invernizzi et al. reported a positive correlation between these two parameters and NV occurrence, suggesting that choroidal vascular changes may precede an upregulation of VEGF and the development of NV [22]. 

Also, CT and CVI variations have been evidenced after IV of anti-VEGF. Temel et al. and Pellegrini et al. found a decrease in CVI in eyes affected by nAMD after anti-VEGF treatment [29,30]. 

Toto et al. found a significant reduction in CT in patients with MNV type 1 after brolucizumab intravitreal treatment [31].

Similar to nAMD, other authors have found CVI changes in other vascular diseases, such as macular telangiectasia type 2, compared to healthy aged-matched controls [32,33].

Particularly, a lower CVI was found in macular telangiectasia type 2 patients compared to healthy subjects. 

Chhablani et al. hypothesized that choroidal thinning with decreased choroidal blood flow could lead to ischemia of the Müller cells, which eventually could cause sprouting of ectatic capillaries [34].

Karasu et al. found lower CVI in non-proliferative and proliferative macular telangiectasia type 2 patients compared to healthy subjects and suggested, as previously claimed in a study by Melrose et al., that subretinal neovascularization in the proliferative macular telangiectasia type 2 may cause choroidal thinning [33,35].

Using structural OCT and OCTA, this is the first study to examine the choroidal and retinal features in eyes affected by different MNV types in nAMD before starting anti-VEGF treatment. Our results could be important for better understanding the nAMD pathogenesis and for distinguishing the vascular behavior of the different subtypes.

Our study has several limitations that should be considered when interpreting our findings. First of all, the study design was retrospective, and secondly, the sample size was not homogeneous between the different MNV types. 

In conclusion, our study reports significant CVI differences between eyes affected by nAMD, particularly MNV type 1 and normal controls, and between different MNV types. Furthermore, we demonstrated a significant negative correlation between the MNV flow area of different MNV types and CVI, supporting the hypothesis of a relationship between reduced choroidal vascularity and MNV flow.

## 5. Conclusions

Choroidal vascularity index was significantly different between patients with MNV and healthy subjects, and showed a negative correlation with the ORCC flow area of all MNV types, suggesting the role of reduced choroidal vascularity in the pathogenesis of multiple diseases.

## Figures and Tables

**Table 1:** Summary descriptive statistics by MNV groups for patients’ demographic characteristics.

Characteristics	MNV Type 1	MNV Type 2	MNV Type 3	*p*-Value
	*n* = 37	*n* = 52	*n* = 16	
Gender, *n* (%)				0.222 ^a^
Female	15 (40.5)	20 (38.5)	10 (62.5)	
Male	22 (59.5)	32 (61.5)	6 (37.5)	
Age (years) median [q1;q3]	75.0 [70.0;80.0]	78.0 [71.0;81.0]	73.0 [69.8;81.0]	0.709 ^b^

^a^*p*-value derived from Chi-squared test; ^b^ Kruskal–Wallis H test.

**Table 2:** Summary descriptive statistics expressed by median value (q1 = first quartile; q3 = third quartile) and absolute frequency (%) by groups of MNV (1, 2, 3) for the ophthalmologic parameters. The *p*-value was derived from Kruskal–Wallis H test.

Variables	MNV Type 1	MNV Type 2	MNV Type 3	*p*-Value
	*n* = 37	*n* = 52	*n* = 16	
CVI	0.63 [0.60;0.65]	0.67 [0.64;0.70]	0.64 [0.63;0.69]	<0.001
Subfoveal CT (μm)	176.00 [142.00;234.00]	172.00 [140.00;212.00]	146.00 [136.00;210.00]	0.458
CMT (μm)	345.00 [319.00;417.00]	442.00 [352.00;547.00]	388.00 [306.00;482.00]	0.006
ORCCFA (mm^2^)	0.18 [0.12;0.22]	0.14 [0.10;0.18]	0.17 [0.12;0.20]	0.032

CVI: choroidal vascularity index; CT: choroidal thickness; CMT: central macular thickness; ORCCFA: outer retina to choriocapillaris flow area.

## Data Availability

Datas are unaivailable due to privacy restrictions.
